# A Typology of the Level of Market Participation among Smallholder Farmers in South Africa: Limpopo and Mpumalanga Provinces

**DOI:** 10.3390/su13147699

**Published:** 2021-07-09

**Authors:** Simphiwe Innocentia Hlatshwayo, Mjabuliseni Ngidi, Temitope Ojo, Albert Thembinkosi Modi, Tafadzwanashe Mabhaudhi, Rob Slotow

**Affiliations:** 1 African Centre for Food Security, School of Agricultural, Earth and Environmental Sciences, College of Agriculture, Engineering and Science, University of KwaZulu-Natal, Private Bag X01, Scottsville, Pietermaritzburg 3201, South Africa; 2 Centre for Transformative Agricultural and Food Systems, School of Agricultural, Earth and Environmental Sciences, College of Agriculture, Engineering and Science, University of KwaZulu-Natal, Private Bag X01, Scottsville, Pietermaritzburg 3201, South Africa; 3 Department of Agricultural Extension and Rural Resource Management, School of Agricultural, Earth and Environmental Sciences, College of Agriculture, Engineering and Science, University of KwaZulu-Natal, Private Bag X01, Scottsville, Pietermaritzburg 3201, South Africa; 4 Department of Agricultural Economics, Obafemi Awolowo University, Ile-Ife 22005, Nigeria; 5 Disaster Management Training and Education Centre for Africa, University of the Free State, Bloemfontein 9301, South Africa; 6 Centre for Transformative Agricultural and Food Systems, School of Life Sciences, College of Agriculture, Engineering and Science, University of KwaZulu-Natal, Private Bag X01, Scottsville, Pietermaritzburg 3201, South Africa

**Keywords:** agriculture, smallholder farmers, market participation, double-hurdle model, policies

## Abstract

Smallholder farmers face several challenges that limit their access to markets and prevent them from taking advantage of market opportunities. This study sought to provide observed information on households’ involvement in the output market and to analyse the determinants of the level of market participation among smallholder farmers in South Africa. Data (secondary) for this study were collected from a total of 1520 respondents who were selected through stratified random sampling. Descriptive statistics, *t*-test and a double-hurdle model were used to analyse factors influencing smallholder farmers’ decisions regarding participation in the agricultural market. The first-hurdle equation of the double-hurdle model showed that gender of the household, family member working on the farm, wealth index, and agricultural assistance had a positive significant impact on the decision of smallholder farmers to participate in the market, while household age and family member with HIV had a negative significant impact. The results of the second-hurdle model showed marital status, educational level of household, wealth index, and access to agricultural assistance had a negative significant effect on the extent of market participation among smallholder farmers, while household size, household age, and family member with HIV had a positive significant impact. The suggestions emanating from the results as to what factors need to be addressed to encourage smallholder farmers to participate in the market indicate that there is a need for government to hire sufficient and skilled extension workers who understand the market related issues. With the help of extension workers and policymakers, government need to organize smallholder farmers into groups that are easy to manage, train, and support. Smallholder farmers’ groups should have their farmers registered, visible, and easily accessible for coordinated government support services. They also need to do more workshops in rural areas to encourage young people to be involved in agriculture. This will lead to sustainable production, alleviation of poverty, improvement of the economy, and food security.

## 1 Introduction

The agricultural sector continues to be strategic in the development of developing nations in Africa, where smallholder farming is the dominant livelihood activity. The agricultural sector in South Africa accounts for around 2.3% of the country’s GDP, 40% of export earnings, and 4.6% of employment in the country [1]. In South Africa, Statistics South Africa (StatSA) [1] showed that 13.8% (2.33 million) of all the households are agricultural households, many of them situated in rural areas. Smallholder agriculture provides about 70% of the employment in rural households and serves as the main source of income [2]. Smallholder agriculture plays a vital role in food security, job creation, and reasonable distribution of income, poverty alleviation, and linkage creation for economic growth [3–5]. The agricultural sector has proven to be the backbone of improvement of rural food security and livelihoods in the country [6]. However, the sector is facing numerous challenges, such as insufficient access to technology, institutional difficulties, inappropriate policies, poor infrastructure, and unsuccessful links to the markets, which make it difficult for smallholder farmers to participate in the formal market sector [7]. In South Africa, there are two types of marketing (formal and informal). Formal marketing involves the formal movement of crops through a different chain of factors, such as seed producers, crop growers, distributors, merchants, and agro-dealers, while informal marketing involves the decentralized market distribution where smallholder farmers sell or exchange crops directly with other farmers, neighbours, or local communities. Smallholder farmers produce traditional crops more for consumption, and they depend on informal markets to sell their surpluses due to inadequate linkages with formal markets [8]. This emphasizes the need to reconsider policies and institutions that support smallholder agricultural participation in the formal markets.

Market participation holds significant potential for revealing suitable opportunity sets necessary for providing better incomes and sustainable livelihoods for smallholder farmers [9–11]. Facilitating the development of market participation by smallholder farmers can be important in helping households to alleviate food poverty and food insecurity [12,13]. It can also enable smallholder farmers to have access to affordable production inputs; hence, this will ensure that farmers are not trapped in low productivity–low return farming activities that lead to food vulnerability. The use of better-quality inputs will improve the ability of smallholder farmers to produce enough marketable surplus and subsequently leads to a better market orientation of goods produced by farmers [14]. The need for developing smallholder market participation has been progressively recognized in efforts to achieve agricultural transformation in developing countries [15]. However, smallholder farmers, particularly in South Africa, are faced with several barriers preventing them from gaining access to markets and productive assets.

Many of the smallholder farmers in South Africa are currently inactive participants, often obliged to sell low (immediately after harvest) and buy high; with little information on where to conduct transactions, they end up being price takers [16]. The constraints affecting smallholder farmers in market participation can be classified as technical, institutional, and socio-demographic factors [16]. Smallholder farmers are living in remote areas with poorly maintained roads and market infrastructure, inadequate transport and storage facilities, and lack of skills and information, which cause high transaction costs of market participation [17,18]. Farmers mainly produce for consumption and sell the surplus to their local communities; the small surplus they produce prevents them from participating in a competitive market and exposes them to high risks and transaction costs that limit them to a non-contestable market dominated by a few powerful buyers [19]. They are faced with incapacities to have contractual agreements, low access to extension agents, poor organizational support, low use of improved seed, and low use of fertilizer, all of which also make it difficult for farmers to commercialize [20]. Other factors that affect farmers’ participation include household size, age and education, source of income, marital status, and human immunodeficiency virus (HIV) status of household members. The impact of HIV on agriculture is very important, as it results in a decline in agricultural production [21]. HIV affects the number of workers available for agricultural activities, leading to low production and productivity, and thus reduces the food stocks that could potentially be taken to the market as part of the outputs for smallholder farmers. The HIV pandemic has an effect on both national development and household economies, which worsens poverty and inequalities [22]. It increases the mortality rate of young and most productive people, which affects smallholder agriculture since it is labour intensive [23]. The epidemic worsens inequalities, poverty, and reduces labour productivity and supply, which slows economic growth [21]. Furthermore, when these conditions get worse, they in turn make households even more at risk and vulnerable to the epidemic. Therefore, it is important to prioritize the improvement of smallholder agriculture to increase the economic activities of smallholder farmers so they can competitively participate in the market. Provision of support by government, policymakers, and other stakeholders can improve the productivity and profitability of smallholder farmers.

Research has been done on market participation in different parts of developing countries such as South Africa. Several studies have been conducted on market participation involving livestock farming, such as cattle and goats [24–26]; other studies considered constraints to market participation [18,27–29]. However, there is limited knowledge on the participation of smallholder farmers in the market within the South African context. It is against this backdrop that this study sought to understand the typology of the level of market participation among the smallholder farmers in South Africa. The study attempted to fill the research gap and contribute to the generation of evidence for policymakers to realize the inequalities that still exist in the market of South Africa and the need to review existing policies. Therefore, the study generated new empirical information on the simultaneous interaction of household decisions of market participation and the most influential factors on the market participation of smallholder farmers in South Africa.

## 2 Literature Review on Factors Affecting Market Participation by Smallholder Farmers in Developing Countries

Marketing is a formalized system that can be directed from seed producer to farmer, or via a chain of actors including distributors, merchants, and agro-dealers until the end user [30]. Marketing aims to identify, anticipate, and satisfy the need of customers and to achieve the objectives of suppliers [31]. To smallholder farmers, marketing means selling or exchanging what they produce on the farm to other farmers, neighbours, or the local community. To a retailer, marketing means promoting goods and services to their consumers. Markets play an important role in production, as they act as a mechanism for exchange. Market participation by smallholder farmers is essential, as it results in the coordination and efficient use of resources, goods, and services [27]. It allows smallholder farmers to derive benefits such as income and accessible opportunities for rural employment [27]. In addition, the involvement of farmers in the market sector can expose rural households to other market activities, such as transportation, processing, and selling, which can employ those who are not willing to participate in the farming sector [29]. In developing countries such as South Africa, market participation can promote sustainable agriculture and economic growth and can also lessen poverty and inequality. Unfortunately, smallholder farmers face difficulties in accessing markets, and as a result, markets fail to effectively perform their duty, which is to provide profits and income to smallholder farmers.

There are several determinants of market participation of smallholder farmers, which can be categorized as institutional, technical, and socio-demographic factors. It is essential to this paper to identify the constraints that smallholder farmers face within market participation. The institutional factors include transaction costs, contractual arrangements, inappropriate policies, and markets information flows. Many studies conducted in the rural economies of developing countries confirmed that smallholder farmers lack adequate market information and contractual arrangements that allow them to formally participate in the market [18,27,28,32]. These factors result in high transaction costs and may cause farmers to either stop participating in the market or lead them to participate in informal markets [32,33]. Jari and Fraser [17] revealed that in the Eastern Cape Province in the South Africa market, information, expertise on grades and standards, contractual agreements, social capital, market infrastructure, group participation, and tradition significantly influenced household marketing behaviour. Many farmers did not participate in the market because they lacked market information, expertise on grade and standards, and contractual agreements. This was substantiated by Sebatta et al. [18], who found that smallholder farmers in Uganda failed to access market information due to remoteness and lack of access to market arrangements, which affected their decision to enter the potato market to sell. The study also found that few farmers were visited by extension officers who provided information on market availability as well as information on new and improved varieties that enhanced the farmer’s knowledge and provided a range and choice of market opportunities. This indicates the level of inequality in service deliveries among farmers in rural areas.

Technical factors are those factors that allow input and output on the market to be accessible at lower costs and that allow diversification of markets. These factors are typically influenced by organization, regulations, and improvements in technology [17]. The factors include market transportation facilities, road infrastructure, household asset holdings, telecommunication networks, storage facilities, and access to extension services. In South Africa, many smallholder farmers live in remote areas where there are limited services, such as poor road infrastructure, storage and transportation facilities, and communication links, and they have limited capacity to add value to their produce [34]. Omiti et al. [28] conducted a study on factors affecting market participation by smallholder farmers in rural and peri-urban areas. The results showed that farmers in peri-urban areas sold higher proportions of their output than those in rural areas. This was because of the distance from farm to the market, poor market information, and poor roads experienced by farmers in the rural areas, which affected their sales. These findings were supported by Zamasiya et al. [35] who found that ownership of radios, television, and cell phones improved access to market information and had a positive effect on the household’s decision to participate in the soybean market in Zimbabwe.

Household asset holdings can help to alleviate any production and market shocks that smallholder farmers experience. Assets such as land, livestock, and human capital and farm implements are crucial for marketable surplus production at a smallholder level [36]. In South Asia and Sub-Saharan Africa, almost 60% of rural households own less than 1 ha of farmland, and approximately 80% of the rural households have less than 2 ha of farmland [37]. Farmers owning small farms may not be able to raise the necessary surplus to sell at the market [38]. South Africa is faced with an increasing population, which causes a decline in per capita farm sizes, especially in rural areas. This influences their ability to feed themselves and their families and to sell the surplus in the market. Smallholder farmers with more land can produce more of the crops, and they can be able to expand their production to ensure sustainable supply to the market. Osmani and Hossain [34] found that smallholder farmers with adequate land, household labour, and farm income had a moderate level of participation in the market, and they had 57% sales in their produced crops. It can be said that holding farm assets can enable smallholder farmers to exercise economies of scale by adopting modern technologies [39].

The socio-demographic factors that affect smallholder farmers’ participation in the markets can include household head age, marital status, household size, source of labour, education, and gender. Farming in rural areas is mostly dominated by women, who are involved in subsistence agriculture. Rural women are an essential resource in agriculture and the rural economy in developing countries [40]. They often manage multifaceted households, provide agricultural labour force, and pursue multiple livelihood strategies. However, female farmers grow subsistence crops mainly for household consumption, and cash crops that are meant to provide income are mainly grown by male farmers [41]. Sebatta et al. [18] showed that females were less likely to participate in the process of selling potatoes in Uganda. Related to that, Vargas Hill and Vigneri [41] found that in Uganda, female farmers sold coffee in the same market as male farmers; however, females significantly got lower prices for the same coffee. Furthermore, Kyaw et al. [42] conducted a study on farmers participation in the rice markets in the central dry zone of Myanmar, the results revealed that 77% of the market participants were male, while 23% were female. On the contrary, Zamasiya et al. [35] found that in Zimbabwe, male-headed households were less likely than female-headed households to participate in soybean markets. The study concluded that this was because legumes are believed to be women’s crops in developing countries. These findings indicate that even in farming there is still gender inequality and that the role of female farmers is underrated.

In South Africa, most of the smallholder farmers are old and not educated; they rely more on their traditions, which make them reluctant to adopt modern technologies that will improve their participation in the market [43]. Participation in the market declines with age, as older farmers are more susceptible to risk aversion and conservative attitudes [44,45]. It can be said that education can empower a farmer to make informed decisions and to be able to identify market opportunities. Sebatta et al. [18] and Adeoti et al. [46] found from their studies that in Uganda and Nigeria, farmers’ educational status showed a positive relationship with market participation. Marital status affects farmers’ market participation differently: Egbetokun and Omonona [47] identified marital status as a major factor that influences participation in markets and reported a positive and significant impact in Nigeria. Contrarily, Adeoye and Adegbite [48] stated a significant but negative effect of marital status on participation in markets. Nwafor [49] found that in Nigeria, 80% of married farmers participated in the market, and 20% did not participate. All the results obtained in the different studies show that more training and workshops need to be conducted in rural areas to increase market participation of farmers, taking into consideration the constraints they are faced with.

All the constraints mentioned above results in high transaction costs, which prevent farmers from getting meaningful benefits from their trading activities, thus discouraging farmers from marketing activities. Smallholder farmers operate under informal production systems, and they depend on traditional social networks and mechanisms for marketing their produce. Most smallholder farmers use barter, traditional labour payment, or gifts to exchange or obtain seeds and crops [50]. Most of their produce exchange takes place within the community, between members within the same social class and ethnic group. Most of the smallholder farmers are price takers and do not have much power to influence market decisions. Monyo and Bänziger [51] stated that more than 90% of farmers’ necessities are met through these informal channels. There is an urgent need to strengthen market information delivery systems, upgrade roads, encourage market integration initiatives, and establish more retail outlets with improved market facilities in the remote rural areas to promote production and trade in high-value commodities by rural farmers. Therefore, analysis of the factors affecting the market participation decision of smallholder farmers will help to design appropriate policy instruments, institutions, and other interventions for their sustainable economic development. It can be concluded that more research is needed to provide evidence-based information for policy and government interventions.

## 3 Research Methodology

The data used in this research were part of a larger baseline assessment study that was conducted in the different provinces in South Africa in 2016 to obtain a comprehensive understanding of livelihood systems and to determine the extent of food and nutrition insecurity. This study focused on two provinces of South Africa (Limpopo and Mpumalanga). The two provinces are populated by smallholder communal farmers who mainly depend on agricultural and livestock farming for their livelihoods. Moreover, the two provinces have relatively more smallholder farmers participating in the market. Although the data were collected in 2016, the findings based on these data are still relevant and very important in improving the situation of the smallholder farmers regarding their participation in the market. The insights drawn from the findings based on these data are still important and critical to enhancing the existing literature. Considering that these data are reported at the household level whilst other national datasets, such as the General Household Survey conducted by Statistics South Africa, are aggregated at a provincial level to form representation, the findings based on these data are relevant for the government and policymakers because the findings can inform policy and programme interventions at a household level. No other comprehensive agricultural, food, and nutrition security dataset that is representative at a household level has been collected in the country so far. In addition, the government, as a custodian of these data, therefore has an interest in what comes out from the data in terms of policy and programme recommendations. Permission to use this dataset was granted by the government, suggesting their willingness to see these data being used to help inform better programming based on evidence.

**Figure 1:**
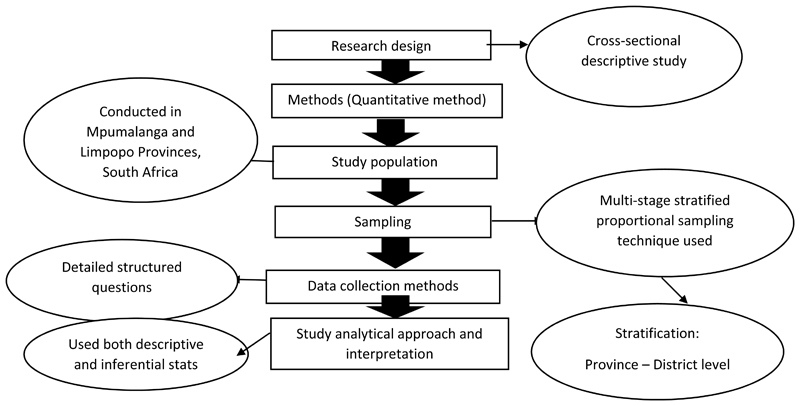
Flow chart of the key components of the research design and procedures. Source: Authors’ own analysis.

The study used a quantitative research method to collect data. Data on key agricultural, food, and nutrition security indicators were collected from a household sample drawn using a multi-stage stratified random sampling technique to collect quantitative information through a survey on 3 districts of Limpopo and 4 districts of Mpumalanga. The multi-stage stratified technique is a random sampling process that allows individuals in a certain population to have an equal and independent chance of being selected [52]. This technique was used because it is quite easy to implement, cheap to use, and it requires the least knowledge of the population to be sampled. It also allows large sampling, as large samples are accurate in the representation of the population, while smaller samples produce less accurate results, and they are likely to be less representative of the population. In each site, the livelihood of the population, including farmers, was divided into strata based on similar characteristics or variables (socio-economic characteristics, outputs, sales, household sizes, and institutional factors). The populations of the Livelihood Zones (geographical areas in which people broadly share similar patterns of livelihoods) produced by the South African Vulnerability Assessment Committee (SAVAC) in 2014 were used as the assessment’s sample frame. Accordingly, the current study used these data as secondary data, which were collected by the SAVAC, led by the Secretariat hosted in the Department of Agriculture, Land Reform, and Rural Development (DALRRD) in 2016. A total of 1520 respondents were selected from two provinces (Limpopo and Mpumalanga). Figure 1 shows a summary of how the research was designed and its procedures.

The data were captured in a computerized manner using Statistical Packages for Social Science (SPSS) (IBM, 2014). The marketing decision of crop farmers was modelled as a two-step decision process: (1) the household decides whether or not to participate in the market and (2) the household decides on the volume of crops to be marketed. The double-hurdle model Cragg [53] cited by Achandi and Mujawamariya [38] was used to model this two-step decision process, following numerous other market participation studies [38,54–58]. This model was chosen over the Heckman sample selection model, which has been used by many studies [18,34,42,45,59]. The Heckman method addresses the statistical challenge posed by cases in which market sales equal zero as a missing data problem. However, when considering the issue of zero market sales, representing a zero amount of maize output sold as a missing value is not a valid economic choice for a model to explain [54]. The double-hurdle model produces estimates that are superior to the Heckman model when one is dealing with true zeros.

According to the double-hurdle model, a farmer faces two hurdles while deciding on market participation: whether or not to participate in the market and how much of their crop to sell in the market. With the assumption of the error terms in the equations being conditionally uncorrelated on all covariates, the standard errors from separate estimations are also valid for conducting statistical inference. If the conditionally uncorrelated errors assumption does not hold, coefficient estimates from separate regressions will be biased [60]. According to Wooldridge [61], testing for conditionally uncorrelated errors follows the same method as does the Heckman test for selection bias. Although it is not technically necessary for identification, it is standard to impose at least one justifiable exclusion restriction when estimating the second stage. The null hypothesis that the first and second stage errors are conditionally uncorrelated is tested using the standard *t*-statistic for the coefficient estimate on inverse mill ration (*IMR)*. If the coefficient estimate is statistically significantly different than zero, we reject the null hypothesis and the model must be re-estimated to conduct valid inference [62]. If we fail to reject the null, we re-estimate second-stage parameters excluding *IMR*.

The variables in the first equation of dependent variables were estimated using the probit model. The probit model accounts for the clustering of zeros due to non-participation, and it is used to predict the probability of whether smallholder farmers participate in the market [45].

The double-hurdle model is stated as:

(1) $$\begin{array}{l} Y_{i}\;=\;\text{0}\;if\;Z_{ni}\;<\;\text{0}\\ Y_{i}\;=\;\text{1}\;if\;Z_{ni}\;>\;\text{0} \end{array}$$

where *Y_i_* is an indicator variable equal to unity for smallholder farmers that participated in the market. *Z_ni_* is the quantity of crop sales made by smallholder farmer *i*

The participation equation can then be written as:

(2) $$Y_{i*}\text{ = }\beta_{\text{1}i}X_{\text{1}i}\text{+}\in_{1i}$$

where Y_i_* is the latent level of utility farmers get from participating in the market, and *∊* is the error term.

The binary model is then stated as:

(3) $$Y\text{ =}\left[\begin{array}{l} \text{1;}\;if\;farmers\;sell\;crops\text{,}\\ 0\;if\;otherwise \end{array}\right]$$

In exact terms, the probit model in stage one of estimation is stated as:

(4) $$P_{r}(Y_{1}\text{) = }\beta_{0}+\beta_{\text{1}}X_{\text{1}}+\beta_{\text{2}}X_{\text{2}}\text{+}......\beta_{n}X_{n}\text{+}\in$$

where, *P_r_*(*Y*_1_) is the probability of a smallholder farmer making a decision to sell crops in the market, *β*_0_ is a constant, *β*_1_ . . . *β_n_* are parameters to be estimated, *X*_1_ …. *X_n_* are the vector of explanatory variables identified in Table 1, and *∊* is an error term.

In the second step, an additional regressor in the sales equation will be included to correct for potential selection bias. This regressor is the inverse Mills ratio (IMR). The IMR is computed as Equation (6):

(5) $$\frac{\phi \left(h\left(\frac{W_{i}}{\alpha }\right)\right)}{\phi (W_{i},\alpha )}$$

where *ϕ* is the normal probability density function. The second-stage equation is given by Equation (7):

(6) $$E=(Y_{i}/Z)=f(x_{i}\beta )+r\frac{\phi \left[h\left(\frac{W_{i}}{\alpha }\right)\right]}{\phi (W_{i},\alpha )}$$

where *E* is the expectation operator, *Y* is the (continuous) proportion of rice sold, *x* is a vector of independent variables affecting the quantity of rice sold, and *β* is the vector of the corresponding coefficients to be estimated. The extent of participation is indicated by:

(7) $$H_{i}\;=\;X_{i}\beta \;+\;V_{i}$$

where *H_i_* is the number of crops marketed, *X_i_* is a vector of covariates that explains this amount, *β* is a vector of unobserved parameters to be estimated, and *V_i_* is a random variable indicating all other factors apart from *X*.

Count data are non-normal and hence are not well estimated by ordinary least squares (OLS) regression [63]. The most common regression models used to analyse count data models include the Poisson regression model (PRM), the negative binomial regression model (NBRM), the zero-inflated Poisson (ZIP), and the zero-inflated negative binomial (ZINB). The PRM and NBRM regression models have become the standard models for the analysis of response variables with non-negative integers [64]. The PRM and ZIP models were used in this study because diagnostic tests revealed the absence of overdispersion and under dispersion. Following Wooldridge [61] and Greene [64], the density function of the Poisson regression model is given by:

(8) $$P_{r}(Y=y)=\frac{\ell^{-\delta (y)}\delta_{i}{\left(Y\right)}^{y}}{\phi (1+Y)}$$

where *δ_i_* = *Exp*(Ω + *L^i^* Ψ) and *Y_i_* = 0, 1 . . . *i* is the number of crops sold by farmers, while *L* is the vector of predictor variables and Ω and Ψ are the parameters to be estimated. Greene (2003; 2008) shows that the expected number of events *δ* (in this case, number of crops sold by farmers) is given as;

(9) $$E(Y=y)=Var(Y_{i}/y_{i})=\sigma_{i}=Exp(\Omega +L\text{Ψ})$$

### Empirical Estimation Procedure and Hypothesis Testing

Estimation of the model outlined above in Equations (1) to (10) followed a series of regression diagnostics. Variables used in both stages of the model were first checked for normality using exploratory data analysis using the coefficient of kurtosis and skewness. To correct for selectivity bias, an inverse Mills ratio (IMR) predicted from the first-hurdle equation was used as a covariate in the count data model (second-hurdle).

## 4 Results

### 4.1 Demographic and Socioeconomic Characteristics of Farm Household in Relation to Market Participation

In the sample of 1520 rural households, 386 were crop producers, and 1134 were non-crop producers. The descriptive analysis revealed that 389 farmers participated in the market and 1131 did not (Table 2).

Tables 3 and 4 show the differences in demographic characteristics between market participants and non-market participants. The *t*-test result showed that the mean age and education were not significant among farmers’ market participation. The mean age of market participants was 1.24 years, while for non-market participants, it was 1.29 years. The mean number of years spent in formal school by market participants was 10.45, while for non-market participants it was 9.32. There were significant differences (*P* < 0.05) in the mean output of crops and market participation. The average yield harvested for market participants was 2242.69, while it was 717 kg for non-market participants. This means that market participants’ farmers had high yields compared with non-market participants, which made them consume and sell the surplus in the market. The intensity of market participation as measured by the number of crops sold among the smallholder farmers is presented in Table 2. The mean crops sold was 2.16, with a standard deviation of 1.27. To analyse the determinants of intensity of market participation of households, the number of crops sold in the market was hypothesised as an outcome variable. The dependent variable is a countable dependent variable, which is measured in number and represents the actual number of crops sold per smallholder farmer in the market.

The results reveal that 77% of market participants were female, while 23% were male. Among non-market participants, 61% were female, and 39% were male. In terms of access to agricultural assistance, 26% of market participants had access to extension officers, while 74% did not have access. Among non-participants, 28% had access to agricultural assistance, while 72% did not have access. Regarding access to market information, 15% of market participants had access, while 85% did not have access. Among non-market participants, 34% had access to information, while 66% did not have access. The results also show that 23% of market participants had livestock, while 77% of market participants had no livestock. In terms of non-market participants, 37% had livestock, while 63% did not own any livestock. Non-market participants had more livestock when compared with market participants. Table 5 shows the different means and standard deviations of all the demographic characteristics of smallholder farmers in Limpopo and Mpumalanga provinces, South Africa.

#### 4.1.1 Factors Influencing the Decision of Smallholder Farmers to Participate in the Market

The results in Table 6 highlight the determinants of market participation among smallholder farmers in the Limpopo and Mpumalanga provinces of South Africa. The first-hurdle equation of the double-hurdle model showed that gender of the household, the salary of household, and agricultural assistance were all significant at the 1% level. Surprisingly, education level and distance to the market did not have a significant impact on the decision of smallholder farmers to participate in the market. Furthermore, these variables had unexpected coefficient signs (negative for education level and positive for distance to the market).

Gender of the household was positively related to the probability of household market participation, and it was statistically significant at 1% level. The result also shows that household age had a negative and significant impact on the farmer’s decision to participate in the market. Access to agricultural assistance showed a positive coefficient and was statistically significant at the 1% level. The results reveal that a family member working on the farm had a positive coefficient and was statistically significant. Having a member in a family that is HIV positive had a negative impact on a farmer’s participation in the market and was significant at the 5% level.

#### 4.1.2 The Determinants of the Market Participation Level of Smallholder Crop Farmers: Count Data Model (Second-Hurdle)

The results for factors influencing the level of market participation among the small-holder farmers are as presented in Table 7. To correct for selectivity bias, an inverse Mills ratio (IMR) predicted from the first-hurdle equation was used as a covariate in the count data model (second-hurdle). The IMR was statistically significant, which shows that bias due to selection was a problem. Since the coefficient was significant, the null hypothesis (no selection bias) is rejected. Hence, using a double-hurdle model for estimating determinants and level of market participation while correcting for a selection bias problem is justified. As depicted in Table 5, estimation of Akaike information criterion (AIC) and Bayesian information criterion (BIC) are important to indicate the better model in analysing count data of the level of market participation of smallholder farmers. In this study, the focus was on two count models—namely, the Poisson regression model and the Zero-Inflated regression model. Starting from the AIC values, the Poisson and Zero-Inflated regression models show 16,060.206 and 16,067.302, respectively. In the same vein, for BIC values, the Poisson and Zero-Inflated regression models reveal 16,040.238 and 16,140.62, respectively. It is clearly shown that AIC value is much smaller in the Poisson regression model as compared with the Zero-inflated Poisson model. In the same vein, the BIC values also corroborate the results of AIC justifying the use of Poisson over the ZIP model with the smaller value. Comparing both observations, from AIC and BIC values, the Poisson regression model fits better in analysing the count data level of market participation of smallholder farmers in the study area. The second-hurdle equation showed that household size, household age, HIV status of a member of a family, and agricultural assistance were all statistically significant.

The results show that marital status had a negative influence on the level of participation of smallholder farmers and was statistically significant at the 5% level. The household size indicated a positive impact on the level of participation of farmers and statistically significant at the 1% level. Unlike in the first-hurdle model, household age showed a positive influence on the level of participation in the market by smallholder farmers and was statistically significant at the 5% level. The effect of educational level on the level of participation in the market by farmers differs in many studies depending on the area of the study. In this study, the result reveals that the educational level of the household had a negative influence on the level of participation in the market by farmers and was significant at the 10% level. This study showed surprising results on the impact of agricultural assistance on the level of participation in the market by farmers. Access to agricultural assistance negatively affected the level of participation in the market, and it was statistically significant at the 1% level.

## 5 Discussion

The objective of the study was to determine the factors that affect the level of market participation among smallholder farmers. The results show that smallholder farmers do not have access to extension services and market information. This is because smallholder farmers live in remote areas where there is poor communication, poor infrastructure, and most of the smallholder farmers are illiterate, and thus, access to market information is hampered. These results are in line with the findings from Aliber and Hall [65], who reported that the South African government suffers from funding constraints which lead to less funding for agricultural support services. The agricultural sector is understaffed by extension officers, and the hired ones do not get adequate training on market issues, so it is impossible for them to provide sufficient market information since they are incompetent. The Department of Agriculture, Land Reform, and Rural Development (DALRRD) [66] reported that they are unaware of the existence of most smallholder farmers; this is because smallholder farmers exist in heterogeneous groups, and they are not formally registered.

### 5.1 Factors Influencing the Decision of Smallholder Farmers to Participate in the Market

The possible explanation for the negative impact on education level might be the fact that the more educated age group is young people, and they are not interested in farming in most cases; they are in other occupations. This was substantiated by Osmani and Hossain [34], who found that young household heads are more motivated to choose and study careers other than farming. The positive effect that distance to the nearest town had on farmer’s decision to participate in the market was also found by Sebatta et al. [18], who concluded that it is easier to access buyers who offer better payment terms in the nearest town than far away from the town. Achandi and Mujawamariya [38] did a study on rice market participation: the results showed a positive relationship and explained that when rice is sold in a market that is further away from the village, it might have low transaction costs for wealthy farmers.

The positive relationship between gender and market participation among smallholder farmers implies that gender plays an important role in agriculture. Sebatta et al. [18] reported that males are more likely to decide to participate in the market and also reported that males decide whether to sell or not and how much. Females are more involved in the production side. This aligns with the study of Vargas Hill and Vigneri [41], who posited that females are mainly involved in subsistence farming, while males grow crops for cash income for the needs of the family.

The negative impact that age had on market participation might arise because small-holder agriculture mainly involves older people who are reluctant to participate in the market because of many factors that include time consumption, transaction costs involved, and the distance to the market. Contrary to these results, other studies found a positive relationship between age and farmers’ market participation [43,49]. Sebatta et al. [18] stated that the decision to participate in the market depends on one’s position in the order of hierarchy in the headship of the family. The older household tends to make a decision that affects the family wellbeing; they sell a higher proportion of their produce in the market.

The results show that agricultural assistance had a positive impact on market participation among smallholder farmers. This could be attributable to the fact that when farmers receive agricultural assistance, especially from the government, they produce more and decide to sell more in the market. They receive market training, inputs, and market information. They also get access to new technologies that create more market opportunities for them to market. These results are in line with Jari and Fraser [17] and Kyaw et al. [42], who found that extension services have a positive and significant influence on market participation by smallholder farmers.

Smallholder farming mainly depends more on family labour than on hired labour, so having a family member working on a farm could lead to optimum production, as responsibilities will be shared among family members. This was substantiated by Egbetokun and Omonona [47], who reported that having more family members working in the farm led to high production and more surplus sold in the market. Knowing the wealth index of a smallholder had indicated a positive and significant impact. This is because when farmers know the resources they possess and their living standard, they tend to utilize what they have and produce effectively.

Having a family member that is HIV positive had a negative impact on a farmer’s participation in the market. This is because as the HIV status of an HIV-positive family member increases, there is an increased likelihood of the farmer not participating in the market as a result of less time allotted for agricultural production. It can also decrease labour since smallholder production depends on family labour for agricultural activities if the HIV member is part of the farming activity. According to the Food and Agriculture Organization (FAO) [67], an increasing number of sick HIV-infected in rural areas threaten survival strategies and food security. Rural households are disadvantaged as they have little access to appropriate information and health services and so are less able to equip themselves with the knowledge to prevent the risks of transmission [67,68].

### 5.2 The Determinants of the Market Participation Level of Smallholder Crop Farmers: Count Data Model (Second-Hurdle)

Marital status had a negative impact on the level of participation of smallholder farmers. These results were similar to those of Adeoye and Adegbite [48], who found a significant but negative effect of marital status on the level of participation. On the contrary, Egbetokun and Omonona [47] identified marital status as a major factor that influences the level of participation in the market.

The positive result of household size in this study was unexpected, as many studies have found a negative influence of household size on the level of participation in the market [13,42,48]. These studies explained that an increase in household size causes farmers to produce more for household production. Omiti et al. [28] explained that large household size is labour inefficient and produces less output, thereby leaving less surplus for sale. However, Egbetokun and Omonona [47] found similar positive results to this study, indicating that most smallholder farmers use family labour for farming activities; therefore, an increase in household size would lead to an increase in farm size cultivation, thereby increasing the amount of farm produce to sell.

Household age showed a positive impact on the level of market participation. This means that older farmers are willing to sell more in the market than young ones. Older farmers tend to make better decisions and to have a greater number of contacts in the market, which enables them to find better markets for their produce. When older people retire from another occupation, they invest their funds into farming, which is why they produce to sell—so that they can keep an inheritance for the future generation. The International Fund for Agricultural Development (IFAD) [69] reported that rural youth lately stated that access to information, lack of credit, and negative perceptions around farming are the leading reasons why most African young people are leaving smallholder agriculture. The high unemployment in rural areas may cause young people to migrate from rural areas in search of better opportunities in urban areas or other countries [68,69].

First, the study estimated the first-stage probit model and predicted an inverse Mills ratio (*IMR)* around the probability of being a market participant. The second stage used the count data estimator that assumed conditionally uncorrelated errors and included *IMR* predicted from the first-hurdle equation as an explanatory variable to correct for selection bias.

In the second-hurdle model, smallholder farmers who participated in the market were old and their retirement funds were used for farming. The most applicable results in South Africa are the ones that were received in the first-hurdle model because the majority of smallholder farmers are old and uneducated.

The possible explanation for negative results on educational level can be that most of the educated people are young people who are not mainly involved in agriculture. Other studies found a positive and significant relationship between educational level and level of participation in the market by farmers [46–48]. These studies explained that the positive relationship showed that the increased level of education of the household makes them gather more information and seek new opportunities in the market for their produce. Education empowers farmers to make an informed decision and detect market opportunities. The authors also added that farmers can be able to combine education and traditional knowledge to produce and sell more.

Agricultural assistance from the government, policymakers, and other stakeholders can enhance production, marketing, and consumption of smallholder farmers and can lead to sustainable production. However, this study found a negative impact. A possible explanation for this might be that some extension workers do not train farmers properly. They sometimes provide farmers with sophisticated technology and inputs without any training. These results were contrary to many other studies: Jari and Fraser [17]; Fischer and Qaim [9]; Sebatta et al. [18]; and Kyaw et al. [42] found a positive and significant relationship. These studies explained that having access to agricultural assistance can provide information on market access and improved varieties that can improve a farmer’s knowledge of production. It can also improve access to technology.

## 6 Conclusions and Recommendations

The involvement of smallholder farmers in marketing can play a critical role in meeting their goals, such as food and nutrition security, poverty alleviation, and sustainable agriculture. This study found that the market participation and sales ratio of smallholder farmers are constrained by numerous factors, such as socioeconomic, market, and institutional factors. Smallholder farmer’s market participation was affected by factors such as education level, the gender of the household, salary of household, and agricultural assistance. The results reveal that household size, household age, and HIV status of a member of a family as well as agricultural assistance, marital status, and educational level had significant influences on the level of market participation.

Agricultural extension and advisory services have a considerable contribution to economic and social development, including the facilitation of smallholder farmer development. This, therefore, suggests that in order to develop smallholder farmers and improve their market participation, there is a need to offer quality extension and advisory services. The government need to improve the performance of agricultural extension services in South Africa. More capable and qualified extension workers need to be hired and trained in marketing so that the marketing of produce is part and parcel of their message delivery in their advisory duties to farmers. Generally, smallholder farmers do not get agricultural assistance and market information because they are not formally registered: they exist in non-homogenous groups, while the Department of Agriculture, Land Reform, and Rural Development is faced with budget constraints. It is recommended that the government, extension workers, and policymakers encourage organized smallholder farmers into groups so that they can help them in large numbers at the same time. When the farmers are organized, the services from the government can be coordinated better, as appointed committee members could be responsible for accessing those services on behalf of the whole group. Government should support the smallholder farmers through the provision of training that is sensitive to the fact that they are generally uneducated; therefore, the information should be packaged in a way that is easy for them to comprehend. To improve smallholder farmers’ production and productivity, the government also need to ensure that their support is timely and well-targeted to those who most need it. Intensive programmes are needed to encourage youth to participate in agriculture, as most young people are literate and can therefore easily grasp the marketing information. Much attention and support need to be given towards women’s involvement in market participation, and women also need to be empowered by the government and other interested stakeholders to fully participate in the decision making related to the price of their produce and where to sell it. More workshops especially for young people and women need to be conducted in rural areas to raise awareness of the importance of agriculture.

In light of these findings, the government and policymakers must revise their agricultural marketing policies and redo policies that will favour the conditions under which smallholder farmers live and operate. Government need to follow-up on policy implementation so that accessibilities to markets and sales of crops can improve.

## 7 Limitations of the Study and Directions for Future Research

Data (secondary) used in this study were collected in 2016, so recent primary data are needed to understand how the situation has changed since the last data collection. This study focused on two provinces of South Africa. There is a need to expand research to cover all provinces, as well as to compare market participation across provinces to draw lessons from one province to another. The current study was conducted to establish the extent of smallholder farmers’ participation in the market. Further studies could be done to assess the effect of farmers’ participation in the market on household food and nutrition security and also on income.

## Figures and Tables

**Table 1:** Factors that are estimated to affect market participation decision.

Variable Name	Variable Definition	Variable Type and Measurement	Hypothesized Effect on Market Participation	Results Received by Sebatta, Mugisha [18]; Kyaw, Ahn [42]
Household age	Age of the household head	In years (continuous)	±	+
Gender of household head	Gender of household head	Dummy (1 = male, 0 = female)	+	−
Marriage status	Marriage status of the household head	Marriage status (dummy)		
Household size	Number of family members	Size of household (continuous)	−	−
Educational level of household	Education level of the household head	Years of education (continuous)	+	+
Livestock	Ownership of livestock	Dummy (1 = yes, 0 = no)		−
Distance to the market	Distance to the market	In kilometer (continuous)	−	−
Market information	Access to market information	Dummy (1 = yes, 0 = no)	+	+
Agricultural assistance	Access to extension service	Dummy (1 = yes, 0 = no)	+	+

Notes: indicates whether the hypothesized effect could be positive or negative, + indicate a positive estimated effect and ™ indicate the negative estimated effect. Source: Authors’ own analysis.

**Table 2:** Demographic characteristics of smallholder farmers in Limpopo and Mpumalanga province, South Africa.

Variables	%	Freq
	Crop production	
Crop producers	25	386
Non-crop producers	75	1134
Overall	100	1520
	Farmers’ participation in the market	
Market participant	12.6	389
Non-market participant	74.4	1131
Overall	100	1520
	Mean	Standard Deviation (SD)
Numbers of crops sold	2.16	1.27

Source: Authors’ own analysis.

**Table 3:** Demographic characteristics of smallholder farmers in Limpopo and Mpumalanga provinces.

Characteristics	Market Participation	Mean	*F* Value	Degrees of Freedom	*p*-Value
Household age	Yes	1.24	1.009	129	0.317
	No	1.29	21.52	
Education of household head	Yes	35.36	0.000	102	0.989
	No	33.41		17.14	
Total output of crops (kg)	Yes	2242.69	25.622	818	0.000 ***
	No	717.17		134.00	

Note: *** Indicate significance at 1% level. Source: Authors’ own analysis.

**Table 4:** Demographic characteristics of smallholder farmers in Limpopo and Mpumalanga provinces, South Africa.

**Variable**	**Market Participant (N = 389)**		**Non-Market Participant (N = 1131)**		**Overall Freq**	
	**%**	**Freq**	**%**	**Freq**	
Gender of household					
Female	77	300	61	688	988
Male	23	89	39	443	532
Access to agricultural assistance					
Yes	26	100	28	318	418
No	74	289	72	813	1102
Access to market information					
Yes	15	60	34	387	447
No	85	329	66	744	1073
Ownership of livestock					
Yes	23	89	37	414	503
No	77	300	63	717	1017

Source: Authors’ own analysis.

**Table 5:** Demographic characteristics of smallholder farmers in Limpopo and Mpumalanga provinces, South Africa.

Variable	Mean	Standard Deviation (SD)
Gender of household head	1.27	0.45
Household age	49.12	11.89
Marital status	4.21	2.44
Household size	4.93	2.71
Educational level of household	33.58	40.30
Ownership of livestock	1.77	0.42
Distance to the market	1.86	1.82
Access to market information	1.94	0.24
Access to agricultural assistance	1.92	0.27
Family member with HIV	0.47	0.79
Family member worked on farm	0.98	0.76
Social grant	1.99	0.73
Irrigation type	1.52	0.50
Total output of crops (kg)	3556.22	88,187.067

Source: Authors’ own analysis.

**Table 6:** Probit results for determinants of market participation of crop farmers (first-hurdle).

Variables	Coef.	St. Err	*p*-Value	Margins	St. Err.	*p*-Value
Household size	0.027	0.051	0.600	0.000	0.001	0.599
Gender of household head	1.034	0.379	0.006 ***	0.015	0.005	0.007 ***
Household age	−0.017	0.010	0.086 *	−0.000	0.000	0.084 *
Educational level of household	−0.244	0.656	0.710	−0.004	0.009	0.710
Family member worked on farm	1.308	0.469	0.005 ***	0.019	0.007	0.005 ***
Social grant	−0.248	0.252	0.325	−0.004	0.004	0.326
WEATHINDEX	1.143	0.274	0.000 ***	0.016	0.004	0.000 ***
Irrigation type	0.361	0.386	0.350	0.005	0.006	0.349
Family member with HIV	1.204	0.565	0.033 **	−0.017	0.008	0.027 **
Distance to the market	0.163	0.494	0.742	0.002	0.007	0.741
Agricultural assistance	2.145	0.573	0.000 ***	0.031	0.008	0.000 ***
Constant	−0.207	0.982	0.833
Mean dependent variable	0.649
pseudo *R*^2^	0.958
Chi-square	1788.386
Prob > chi2	0.000

Note: ***, **, * Indicate significance at 1%, 5%, and 10% level, respectively. Source: Authors’ own analysis.

**Table 7:** Determinants of the level of market participation of smallholder crop farmers: count data model (second-hurdle).

	Poisson Regression			Zero-Inflated Poisson Regression		
Number of Crops Sold	Coef.	St. Err	*p*-Value	Coef.	St. Err.	*p*-Value
IMR	0.135	0.062	0.030 **	0.135	0.062	0.030 **
Marital status	−0.192	0.086	0.025 **	−0.192	0.086	0.025 **
Household size	0.009	0.003	0.001 ***	0.009	0.003	0.001 ***
Gender of household	−0.025	0.035	0.469	−0.025	0.035	0.469
Household age	0.001	0.000	0.012 **	0.001	0.000	0.012 ***
Education level of household	−0.141	0.077	0.066 *	−0.141	0.077	0.066 *
Family member worked on farm	0.008	0.070	0.907	0.008	0.070	0.907
Irrigation type	0.085	0.064	0.184	0.085	0.064	0.184
Family member with HIV	0.580	0.082	0.000 ***	0.580	0.082	0.000 ***
Distance to the market	−0.110	0.088	0.212	−0.110	0.088	0.212
Agricultural assistance	−0.073	0.024	0.002 ***	−0.073	0.024	0.002 ***
WEATHINDEX	−0.089	0.037	0.017 **	−0.089	0.037	0.017 **
Social grant	0.033	0.039	0.408	0.033	0.039	0.408
Constant	2.324	0.147	0.000 ***	2.324	0.147	0.000 ***
If the household reside				−7471.85		
Constant				−7947.60		
Mean dependent variable	13.516			13.516
pseudo *R*^2^	0.008			1400.000
Chi-square	122.279			122.279
Number of obs	1400.000			1400.000
Prob > chi2	0.000			0.000
Akaike crit. (AIC)	16,060.206			16,067.30
Bayesian crit. (BIC)	16,040.238			16,140.62

Note: ***, **, * Indicate significance at 1%, 5%, and 10% level, respectively. Source: Authors’ own analysis.

## Data Availability

Restriction apply to the availability of these data. Data was obtained from the Department of Agriculture, Land Reform, and Rural Development (DALRRD) and are available from South African Vulnerability Assessment Committee (SAVAC) secretariat with the permission of Department of Agriculture, Land Reform, and Rural Development (DALRRD).
